# Right atrial volume by cardiovascular magnetic resonance predicts mortality in patients with heart failure with reduced ejection fraction

**DOI:** 10.1371/journal.pone.0173245

**Published:** 2017-04-03

**Authors:** Alexander Ivanov, Ambreen Mohamed, Ahmed Asfour, Jean Ho, Saadat A. Khan, Onn Chen, Igor Klem, Kumudha Ramasubbu, Sorin J. Brener, John F. Heitner

**Affiliations:** 1 Department of Medicine, New York-Presbyterian Brooklyn Methodist Hospital, Brooklyn, New York, United States of America; 2 Department of Medicine, Maimonides Medical Center, Brooklyn, New York, United States of America; 3 Department of Medicine, Duke University Medical Center, Durham, North Carolina, United States of America; Indiana University, UNITED STATES

## Abstract

**Background:**

Right Atrial Volume Index (RAVI) measured by echocardiography is an independent predictor of morbidity in patients with heart failure (HF) with reduced ejection fraction (HFrEF). The aim of this study is to evaluate the predictive value of RAVI assessed by cardiac magnetic resonance (CMR) for all-cause mortality in patients with HFrEF and to assess its additive contribution to the validated Meta-Analysis Global Group in Chronic heart failure (MAGGIC) score.

**Methods and results:**

We identified 243 patients (mean age 60 ± 15; 33% women) with left ventricular ejection fraction (LVEF) ≤ 35% measured by CMR. Right atrial volume was calculated based on area in two- and four -chamber views using validated equation, followed by indexing to body surface area. MAGGIC score was calculated using online calculator. During mean period of 2.4 years 33 patients (14%) died. The mean RAVI was 53 ± 26 ml/m^2^; significantly larger in patients with than without an event (78.7±29 ml/m^2^ vs. 48±22 ml/m^2^, p<0.001). RAVI (per ml/m^2^) was an independent predictor of mortality [HR = 1.03 (1.01–1.04), p = 0.001]. RAVI has a greater discriminatory ability than LVEF, left atrial volume index and right ventricular ejection fraction (RVEF) (C-statistic 0.8±0.08 vs 0.55±0.1, 0.62±0.11, 0.68±0.11, respectively, all p<0.02). The addition of RAVI to the MAGGIC score significantly improves risk stratification (integrated discrimination improvement 13%, and category-free net reclassification improvement 73%, both p<0.001).

**Conclusion:**

RAVI by CMR is an independent predictor of mortality in patients with HFrEF. The addition of RAVI to MAGGIC score improves mortality risk stratification.

## Background

An estimated 5.7 million Americans have heart failure (HF). The foreseen increase in the prevalence of HF will top 8 million by 2030. Approximately 870, 000 new cases of HF are diagnosed annually [1]. Pocock et al. recently published the Meta-Analysis Global Group in Chronic Heart Failure (MAGGIC) score, a uniquely robust and generalizable tool to quantify individual patients’ prognosis in HF[2]. This risk score was developed based on the largest patient dataset available to date. However, the score was developed using historical and clinical patient data, and currently used HF biomarkers and volumetric chamber measurement that have been shown to predict adverse events in HF were not included in the MAGGIC (integer) score.

Right atrium volume index (RAVI) measured by trans-thoracic echocardiography (TTE) was identified as an independent predictor of adverse outcome in patients with HF with reduced ejection fraction (HFrEF) [3]. These findings, however, were observed in a small population and adverse outcomes were driven primarily by readmission rates for HF. It is important to acknowledge that this study used RAVI as an “easy to measure” surrogate of right ventricular (RV) function since reproducible quantifiable RV assessment by TTE is limited. Cardiac magnetic resonance imaging (CMR) provides excellent spatial resolution as well as high reproducibility and provides more accurate volumetric assessment than TTE [4–6].With the recent publication of the standardized approach to measure RAVI by CMR [7], we aimed to to evaluate RAVI as an independent predictor of all-cause mortality, compare discriminatory ability of CMR volumetric parameters as mortality predictors in patients with HFrEF and to assess the added value of those parameters to the MAGGIC score

## Methods

### Protocol

This study is part of an ongoing outcomes registry of patients undergoing CMR imaging at the New York Methodist Hospital. Our study was approved by the institutional review board. Every patient enrolled in this study provided written informed consent for inclusion of CMR, demographic, and outcomes data to the registry. There was no external funding used to support this work. The authors are fully responsible for the design and conduct of this study, all data analysis, drafting, editing of the paper and its final content.

We systematically obtained clinical, demographic, electrocardiographic (baseline rhythm, PR, QRS, QT, QTc intervals as well as presence of LBBB/RBBB) and laboratory data (Na,Creatinine, C-reactive protein and Pro-BNP-NT) via direct patient interview at the time of enrollment in the registry, and review of notes from referring physicians and electronic medical record at the time of CMR scan. Vital status was followed at regular intervals after initial CMR. Data were collected at regular intervals by cardiovascular research associates blinded to the CMR results through either standardized telephone interview with the patients or, if deceased, with family members or contact with the referring physician; review of inpatient and outpatient medical records. Vital status and date of death was additionally confirmed using Social Security Death Index. The primary outcome was all-cause mortality. Cause of death was adjudicated using electronic health records, death certificate, telephone interview with a relative or with a physician involved in care. We defined cause of death as cardiac or non-cardiac.

### Patient population

Patients referred for CMR from June 2006 to December 2014, older than 18 years of age, with severely reduced left ventricular systolic function defined as an ejection fraction (EF) ≤ 35% at index CMR exam were and enrolled in the registry were enrolled in this study. We excluded patients with complex congenital heart disease, prior valve surgery, severe valvular disease by CMR, those scheduled for open heart surgery or patients with inadequate/incomplete imaging.

### Cardiac magnetic resonance imaging

#### Imaging protocol

CMR studies were performed with a 1.5-T CMR system Magnetom Avanto™(Siemens Healthcare®) using standard pulse sequences. Before contrast administration, short-axis steady-state free precession (SSFP) images covering the left ventricle (LV) from the mitral valve annulus to the apex were obtained for the evaluation of LV function. Three chamber, four chamber and two (both right and left sided) chamber SSFP images were also acquired according to the American Heart Association imaging recommendation [8]. All patients received gadolinium based contrast and based on current recommendation for late gadolinium enhancement imaging (LGE) IPAT segmented images and single shots images were obtained. LGE images were analyzed and patients were divided into 2 groups based on LGE patterns: ischemic cardiomyopathy was defined as a presence of subendocardial LGE in the coronary artery distribution or known obstructive coronary artery disease or history of revascularization; non-ischemic- all other patients.

#### Right atrium volume measurement

Two physicians blinded to patient clinical data performed area measurements of the right atrium in the 4-chamber view and right-sided-2 -chamber view at the end systole utilizing retrospectively gated SSFP images. Precession™ software (Heart Imaging Technologies®) was used for area measurements. The right atrial appendage was not included in the measurements. The equation(RA volume = 3.08*(2C area) +3.36*(4C area) -44.4) published elsewhere was utilized to calculate right atrial volume (7). Body surface area (BSA) was calculated according to the Mosteller formula[9]. Right atrial volume was indexed to BSA to obtain RAVI. Since right atrial appendage was not well vizualised in 37% of the patients- decision was made to exclude it from tracing in all patients for higher homogeneity.

#### Left and right ventricular ejection fraction and left atrial volume index measurement

Both left and right ventricular volumes were measured during diastole and systole using PRECESSION software™. For the volumetric assessment of the both ventricles and calculation of ejection fraction we utilized technique described by Bourantas et al [10]. Left Atrial Volume was measured using area length method [11] and then referenced to BSA.

### MAGGIC risk calculation

MAGGIC score was calculated using an on-line calculator (available at http://www.heartfailurerisk.org/). In order to calculate the MAGGIC score data regarding age, sex, history of diabetes, history of chronic obstructive pulmonary disease, duration of heart failure (more or less than 18 months), smoking status, current HF NYHA class[12], treatment with beta-blockers, angiotensin-converting enzyme inhibitors/angiotensin receptor blockers, body mass index, systolic blood pressure, creatinine (μmol/L) and left ventricular ejection fraction were obtained at the time of CMR exam and then subsequently used for calculation of integer score. The integer score with corresponding risk of dying within one and three years according to it were recorded.

### Statistical analysis

We present continuous data as mean, and standard deviation (SD) for normally distributed variables, or median and interquartile range (IQR) for non-gaussian distributed variables. We present categorical data as frequencies. Continuous variables were compared using Mann-Whitney test. The Wilcoxon rank sum-test test was performed for categorical variables. Spearman’s and Pearsons correlation coefficients were used to show a correlation between RAVI and tested variables. The primary outcome was assessed using Cox proportional hazard model controlled for MAGGIC (Integer) risk and right ventricular ejection fraction (RVEF). The proportional hazard assumption was confirmed using Schoenfeld residuals (p for RAVI = 0.847, p for global test = 0.879) and by the scaled Schoenfeld residuals plot. Kaplan–Meier curves with the log-rank statistic were used to illustrate outcome. Receiver operating characteristic (ROC) analysis was used to compare area under the curve (AUC) of RAVI to volumetric CMR parameters (right ventricular ejection fraction, left ventricular ejection fraction and left atrium volume index) and MAGGIC risk for the prediction of mortality [13]. We then used an integrated discriminatory ability (IDI) and category-free net reclassification indexes (NRI) to better quantify the risk reclassification of RAVI when added to MAGGIC risk and volumetric CMR parameters [14–17]. We used Bland Altman method and Spearman correlation to assess variability in measurements. A p-value of <0.05 was considered statistically significant. All calculations were performed using STATA 14.0 (StataCorp, Texas, USA) software.

## Results

### Baseline characteristics

**Fig 1:**
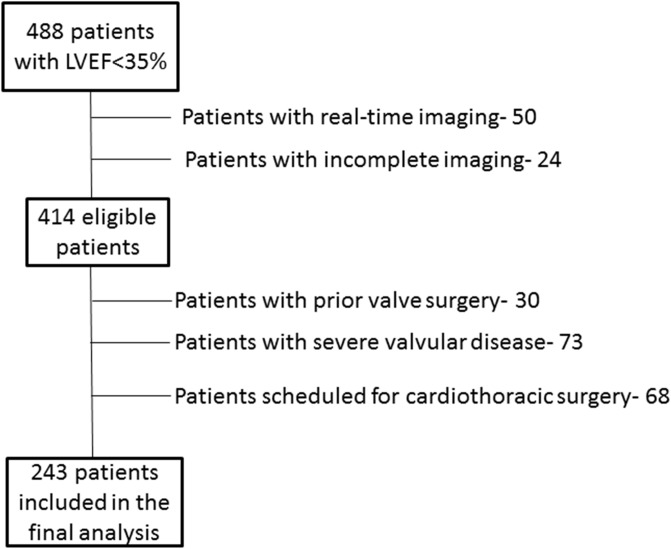
Patient flow chart.

We included 243 patients in our analysis (Fig 1). Baseline demographics, risk factors, medications, laboratory values, CMR data and MAGGIC score are summarized in Table 1. Right atrial volume index was normally distributed in the studied population with median of 44.5 (interquartile range 33.2 to 66.6) ml/m2 and mean 52.3 ± 25.3 ml/m2. MAGGIC risk (integer score) was calculated for 233 patients, and it had a normal distribution- median of 19 (interquartile range 16–24 units) and mean 19.9 ± 6.5 units. According to the MAGGIC score in our cohort, the predicted mortality was 9% at 1 year and 23% at 3 years. There was a significant positive association between RAVI and patient’s age, NYHA class at the time of CMR, atrial fibrillation, B-blocker therapy, pro-BNP level, creatinine, length of PR and QTc interval, diagnosis of amyloidosis by CMR as well as severity of tricuspid regurgitation and MAGGIC score. RAVI was negatively associated with, active smoking, LVEF, RVEF. Of interest, ischemic cardiomyopathy by CMR was positively correlated, and non-ischemic cardiomyopathy by CMR was negatively correlated with RAVI with very similar correlation coefficients and level of significance.

**Table 1:** Baseline characteristics.

Parameter	Allpatients	RAVI ≤44.5 ml/m^2^	RAVI >44.5 ml/m^2^	P
***Nofpatients***	**243**	**122**	**121**	
***Demographics***				
Age-yr*	60±15.6	58.1±14.9	61.7±16.1	0.0632
FemaleGender	79(33%)	38(31.2%)	41(33.9%)	0.65
***Riskfactors***				
BMI (kg/m^2^)	28.6±6.4	28.9±6.4	28.2±6.4	0.3
Hypertension	181(75%)	88(74%)	93(77%)	0.6
Hyperlipidemia	142(59%)	75(62%)	67(55%)	0.3
Diabetes	81(34%)	46(38%)	35(29%)	0.12
CAD	103(43%)	54(45%)	49(41%)	0.52
PriorMI	71(29%)	38(32%)	33(27%)	0.49
NYHAIclass	54(22%)	29(24%)	25(21%)	0.0213
IIclass	105(43%)	63(51.6%)	43(35.3%)	
III class	74(31%)	24(19.7%)	50(41%)	
IVclass	10(4%)	6(5%)	4(3.3%)	
HistoryofAF	38(16%)	8(7%)	30(25%)	0.0001
PAD	11(5%)	6(5%)	5(4%)	0.74
Activesmoking	31(13%)	26(22%)	5(4%)	0.0001
COPD	20(8%)	10(9%)	10(8%)	0.94
Cancer	22(9%)	10(8%)	12(10%)	0.64
PriorCVA	18(7%)	6(45%)	12(10%)	0.15
***Medicationsused***				
RASblocker	154(66%)	83(68%)	89(72.9%)	0.4
Beta-blockers	180(76%)	90(74%)	111(91%)	0.003
MRA	46(20%)	20(17%)	32(26%)	0.1
Otherdiuretics	112(48%)	47(39.2%)	65(54.1%)	0.055
Digoxin	24(10%)	10(9%)	14(12%)	0.5
Statins	125(53%)	63(56%)	62(51%)	0.5
Aspirin	124(53%)	66(58%)	59(48.4%)	0.13
***Laboratoryvalues***				
ProBNP-NT,pg/dl^#^	2139(472;6138)	660(268;2862)	4894(1763;11360)	0.0001
Creatinine,mg/dl	1.22±0.8	1.1±0.6	1.2±0.9	0.004
Na	139.1±3.9	139±4	139±4	0.11
CRP^#^	3.3(1.6;10.7)	2.9(1;6.4)	4.1(2.4;17.1)	0.017
***ECG***				
Sinusrhythm	183(87%)	101(98%)	83(78%)	0.0001
Atrialfibrillation	26(13%)	2(2%)	24(22%)	
PR,ms	170.4±37.4	163.8±32.7	178.6±4.6	0.0008
QRS,ms	104.7±24.1	104.3±25.6	105±22.7	0.5971
QT	411±47.6	407±45	415.2±49.8	0.1237
QTc	466.3±40.4	458.9±39.7	473.6±39.7	0.0097
RBBB	12(6%)	4(4%)	8(8%)	0.263
LBBB	26(12%)	16(16%)	10(10%)	0.175
***CMRscan***				
***reasonforCMRevaluationevaluation***				0.054
Cardiomyopathy	183(75%)	86(70%)	97(80%)	
StressTest-	37(15%)	25(21%)	12(10%)	
Mass/thrombus	19(8%)	7(6%)	12(10%)	
Arrythmia/syncope	5(2%)	4(3%)	1(1%)	
**EtiologyofHF**				
NICM	152(63%)	67(55%)	86(70.5%)	0.014
ICM	87(36%)	52(43%)	35(29%)	0.026
Combined	4(2%)	3(2%)	1(1%)	0.32
AmyloidosisDx	12(5%)	0	12(10.%)	0.0001
***Volumetricanalysis***				
LVEF,%	25(20;32)	26(20;33)	23(18;30)	0.006
LVEDVi	86±36	79±36	94±34	0.0001
LVESVi	66±31	60±33	72±27	0.0001
LVSVi	21±9	20±6	22±11	0.32
RVEF,%	33±11	36±10	29±11	0.0001
RVEDVi	72±26	60±19	84±26	0.0001
RVESVi	50±23	40±17	61±25	0.0001
RVSVi	22±8	21±7	23±9	0.04
LAVI	54±24	41±16	67±23	0.0001
***TRbyCMR***				
None	128(52%)	86(70%)	42(34%)	0.0001
Present	116(48%)	37(30%)	79(66%)	0.0001
***MAGGICrisk***				
Integerscore	19.9±6.5	19±6.1	20.8±6.7	0.03

BMI = Body mass index, CAD = coronary artery disease, Prior MI = prior myocardial infarction, AF = atrial fibrilation, PAD = peripheral artery disease, COPD = chronic obstructive pulmonary disease, CVA = cerebrovascular accident, RAS = renin angiotensin system, MRA = mineralocorticoid antagonist, Pro BNP = pro–B-type natriuretic peptide, RBBB = right bundle brunch block, LBBB = left bundle brunch block, Stress Test = regadenosin perfusion imaging, HF = heart failure, NICM = non-ischemic cardiomyopathy, ICM = ischemic cardiomyopathy, Dx = diagnosis, LVEF = left ventricular ejection fraction, LVEDVi = left ventricular end diastolic volume indexed (ml/m2), LVSVi = left ventricular stroke volume indexed, RVEF = right ventricular ejection fraction, RVEDVi = right ventricular end diastolic volume indexed, RVSVi = right ventricular stroke volume indexed, LAVI = left atrial volume indexed, TR = tricuspid regurgitation, CMR = Cardiovascular Magnetic Resonance, MAGGIC = Meta-analysis Global Group in Chronic Heart Failure

*Plus-minus values are means ± SD, nominal variables presented as N (%)

^#^ Variables are presented as median (interquartile range)

### Right atrial volume and all-cause mortality

**Fig 2:**
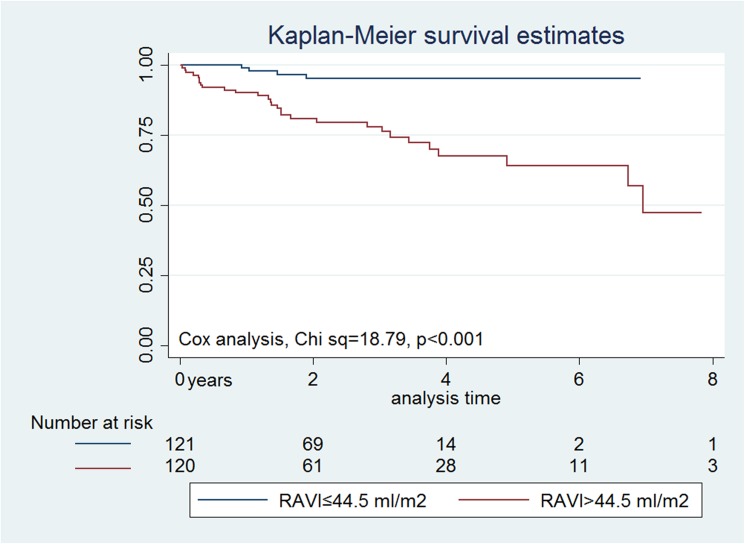
Mortality estimates by RAVI more or less than the median at the time of CMR imaging.

There were 33 deaths (13.6%) over a median follow-up of 2.2 years (interquartile range 1–3.4), and mean follow-up of 2.4 ± 2 years. Of those, seventeen patients (51%) had a cardiac, eight patients (24.5%)- non-cardiac and eight patients (24.5%) had unknown cause of death. There were four and 29 deaths in patients with RAVI ≤ than median and > than median, respectively, P<0.001, corresponding to annual mortality of 9.2% vs. 1.4%, respectively (Fig 2).On average, deceased patients had a higher mean RAVI 78 ml/m ^2^ vs 48 ml/m ^2^, p<0.001. (Fig A in S1 File).Multivariable analysis showed that for every increase in 1 ml/m2 in RAVI, mortality increased by approximately 3% (HR 1.03; 95%CI 1.01–1.04; p<0.001)(Fig B in S1 File), and every unit increase in MAGGIC score carries a nearly 13% increase in mortality (HR 1.13; 95%CI 1.06–1.2; p<0.001). RVEF was not an independent predictor of mortality (p = 0.3, Table 2).

**Table 2:** Multivariable Cox Proportional Hazard Model.

Predictor	HR	95% CI	P
RAVI	1.03	1.01–1.04	0.001
RVEF	1.02	0.98–1.05	0.3
MAGGIC	1.13	1.06–1.2	0.001

RAVI- right atrium volume index

RVEF- right ventricular ejection fraction

MAGGIC- Meta-analysis Global Group in Chronic Heart Failure (Integer score)

**Fig 3:**
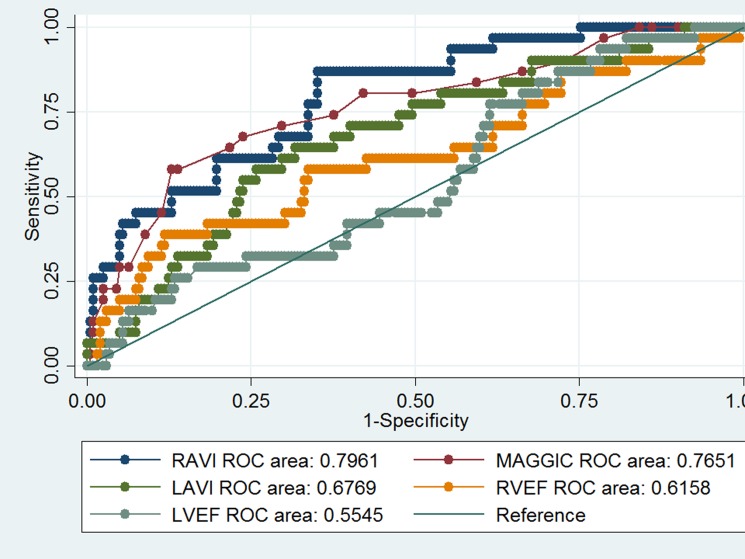
ROC analysis.

Further analysis showed that RAVI has greater C statistics (AUC 0.79 (CI 0.72–0.88) than RVEF (AUC 0.62 (CI 0.50–0.73), p<0.03), LAVI (AUC 0.68 (CI 0.57–0.78), p<0.02) and LVEF (AUC 0.55 (CI 0.45–0.66), p<0.01). There was no difference in C statistic between RAVI and MAGGIC score (AUC 0.77 (CI 0.67–0.86), p = 0.61, Table 3 and Fig 3). However, adding RAVI to the Integer score significantly improved risk stratification (integrated discrimination improvement 13%, p<0.01 and category-free net re-classification improvement 73%, p = p<0.01) (Table 3).

**Table 3:** Discrimination analysis.

Outcome	ROC	SD	P^*^	IDI	P^^^	NRI	P^#^
RAVI	0.80	0.72–0.88					
LAVI	0.68	0.57–0.78	0.018	0.55	<0.0001	0.71	<0.0001
RVEF	0.62	0.50–0.73	0. 027	0.17	<0.0001	0.71	<0.0001
LVEF	0.55	0.45–0.66	0.001	0.16	<0.0001	0.89	<0.0001
MAGGIC	0.77	0.67–0.86	0.61	0.13	0.001	0.73	0.0002

^*^- when compared to RAVI ROC for mortality

^^-^ integrated discrimination improvement when RAVI added

^#^- category free net reclassification improvement when RAVI added

RAVI- right atrium volume index

LAVI-left atrium volume index

RVEF- right ventricular ejection fraction

LVEF-left ventricular ejection fraction

MAGGIC- Meta-analysis Global Group in Chronic Heart Failure (Integer score)

### Reproducibility of right atrial volume measurements

Twenty-five RAVI measurements were randomly selected and measured separately by two operators blinded to each other’s results (A.I. and A.M.).There was a excellent reproducibility of RAVI measurements for both observers: 1.9 ml/m2 (95% CI (-3.2)-4.9) for observer I and 1.5 ml/m2 ((-1.9)- 4.1) for observer II, with mean interobserver RAVI difference assessed by Bland-Altman method was -1.7 ml/m2 (95% CI -2.5–4.4).Spearman correlation coefficient for interobserver variability of RAVI was 0.951 (p<0.001)

## Discussion

We report several findings emphasizing the prognostic importance of RAVI in our cohort of patients with severely reduced left ventricular systolic function referred for CMR imaging. First, RAVI was an independent predictor of mortality even after adjusting for MAGGIC score and right ventricular ejection fraction. Second, RAVI is a stronger predictor of mortality than LVEF and LAVI. Thirdly, addition of RAVI increased mortality discrimination of MAGGIC score when assessed by IDI and NRI. These findings make RAVI an important predictor of mortality in patients with HFrEF and suggest that it provides more important prognostic data when compared to conventionally used predictors.

MAGGIC score is a result of a patient level meta-analysis of 39,372 patients from 30 studies with a median follow-up of 2.5 years—the largest dataset published for score derivation (2). Median and mean integer score were 19 and 20, respectively, in our cohort, consistent with the observed mortality of 12.8% at a median follow-up of 1.6 years. RAVI and MAGGIC score were predictors of mortality in the multivariable analysis, independent of each other. MAGGIC score has excellent discrimination ability for mortality, with AUC of 0.76± 0.1. The addition of RAVI to this well calibrated risk score leads to significant risk reclassification when assessed by IDI and NRI.

Sallach et al.(3) assessed RAVI as a surrogate of right ventricular function by TTE in 192 patients. They found RAVI to be an independent predictor of primary outcome that consisted of mortality, cardiac transplant and repeated hospitalization for HF. In our analysis RVEF was a predictor of mortality only in univariate analysis, and RAVI remains to be an independent predictor in multivariate analysis even after controlling for RVEF and other variables. Moreover RAVI has a greater C statistics than RVEF for predicting mortality. These findings suggest that right atrial volume is a more sensitive parameter than right ventricular systolic function to identify higher risk patients. Their reported mean RAVI was 28 ml/m2, much smaller than in our analysis (53 ml/m2). This difference might be explained by several factors: CMR has a greater precision with higher spatial resolution of endocardial borders, use of slightly different anatomical views to measure right atrium by echocardiography and absence of geometric assumptions in retrospectively gated CMR images in contrast to echocardiography. Whitlock et al. reported a significant underestimation of RA volume by echocardiographic area length method when compared to CMR imaging[18].

There are only a few peer-review articles addressing right atrial dimensions by CMR [7, 19–21]. Those publications differ greatly in imaging sequences, imaging analysis and population used for analysis. We used a method of measuring RAVI by CMR reported by Maceira et al[7]. This method uses area measurement in two- and four-chamber views to calculate right atrial volume. Mean RAVI reported in healthy volunteers was 55± 19 ml/m2, which was similar to the one in our dataset. Most plausible explanation for this similarity in results between healthy volunteers and patients with severe chronic heart failure would be the fact that we didn’t include right atrial appendage in our area measurements.

The underlying pathophysiologic mechanism linking RAVI with mortality is not fully understood. One of the possible explanation maybe the fact that right atrium has thin walls, and it is influenced by the same factors that are affecting right ventricular diastolic filling and increase in RAVI may serve as an early manifestation of right ventricular diastolic dysfunction even when RVEF is still relatively preserved. Further investigation of RAVI’s dynamic changes in patients with heart failure and their correlation with adverse clinical outcomes is warranted.

There were multiple publication elaborating on utilization of transthoracic echocardiography for an evaluation of right ventricular function in patients with HfrEF [22–25]. In one of the series of Damy et al [22] analyzed 1547 patients using tricuspid annular plane systolic excursion (TAPSE) measured by TTE as a surrogate of RVEF in patients with clinical symptoms of HF and found it to be an independent predictor of adverse outcomes. In our paper we use direct RVEF measurement by CMR–which is considered to be the “gold standard” for the right ventricular assessment[26]. Moreover in our multivariate analysis RAVI remains independent predictor of mortality being controlled for RVEF and all clinical information summarized by MAGGIC risk. Additionally our NRI and IDI analysis shows incremental increase in discrimination power when RAVI was added to RVEF.

We recognize several limitations of our study. There is, probably, some degree of referral and selection bias for patients undergoing cardiac MRI in our center. However the external validity of our findings was supported by MAGGIC score—predicted mortality rate is in line with observed one. The total number of patients was significantly affected by our inclusion/exclusion criteria, however our sample size is comparable or exceeding number of patients with LVEF<35% in recent publications[27–29]. Moreover it is the one of the largest analysis with volumetric assesment of all cardiac chambers by any imaging modality (TTE, CT, CMR) in patients with LVEF<35%. We did not include right atrial appendage into right atrial volume measurement since it wasn’t consistently visualized in more than a third of all studied population. Echocardiographic as well as invasive hemodynamic measurements on right ventricular systolic and diastolic pressures were not available for analysis. We did not use a control group of patients with normal LVEF. MAGGIC score is most applicable for patients with stable heart failure as the calculations represent a “snapshot” and can be altered by a decompensated state of disease.

## Conclusion

Despite these limitations, we conclude that right atrium volume index measured by cardiac magnetic resonance imaging is an independent predictor of mortality in patients with heart failure with reduced ejection fraction. The addition of right atrium volume to the MAGGIC score significantly improves mortality risk stratification.

## Supporting information

S1 File Contains Fig A, Fig B.
(DOCX)
