# Correction: Right atrial volume by cardiovascular magnetic resonance predicts mortality in patients with heart failure with reduced ejection fraction

**DOI:** 10.1371/journal.pone.0176942

**Published:** 2017-04-27

**Authors:** The PLOS ONE Staff

The sixth author’s first name is spelled incorrectly. It should read On Chen. The publisher apologizes for the error.
