# Supplementary material for: Right atrial volume by cardiovascular magnetic resonance predicts mortality in patients with heart failure with reduced ejection fraction
Source: PLoS One. 2017 Apr 3;12(4):e0173245. doi: 10.1371/journal.pone.0173245 (PMC5378325; doi:10.1371/journal.pone.0173245)

**Supplementary figures**

**S1 Fig A. Frequency distribution of right atrium volume indexed in patients with and without study outcome.**

**S1 Fig B. Mortality Risk per Adjusted Right Atrium Volume Indexed.**

**S1 Fig A. Frequency distribution of right atrium volume indexed in patients with and without study outcome.**
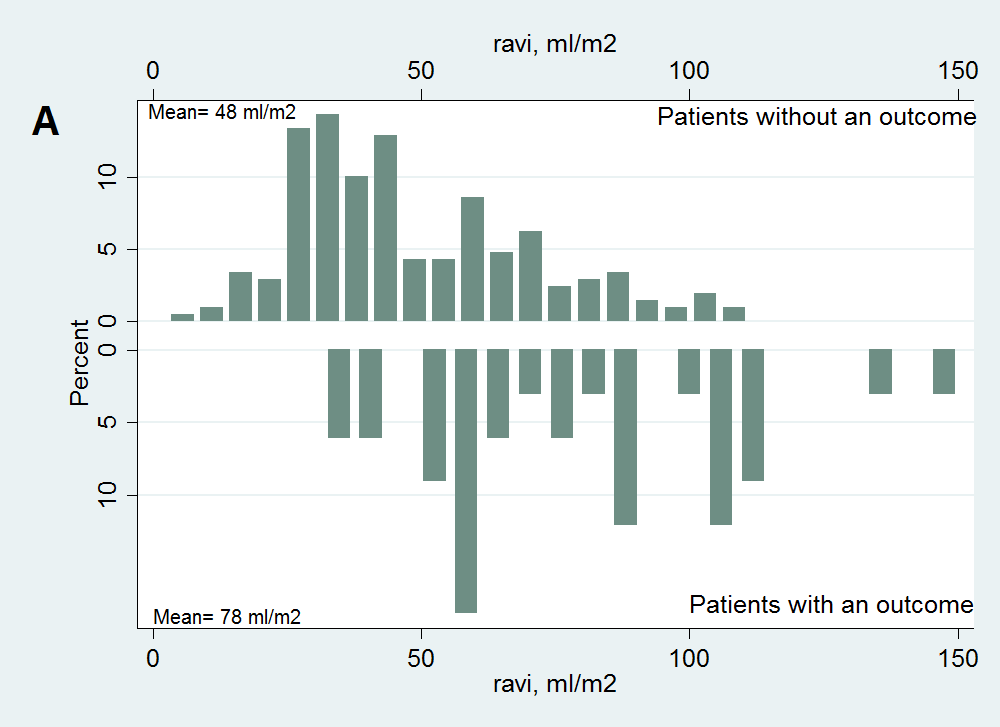


**S1 Fig B. Mortality Risk per Adjusted Right Atrium Volume Indexed.**


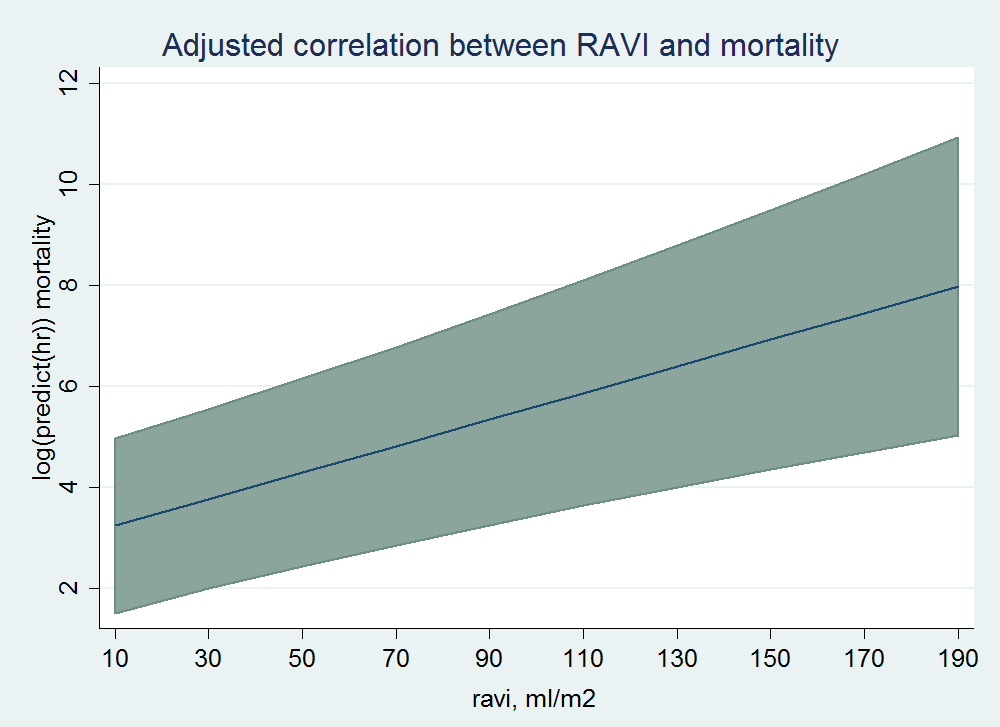

Supplement: S1 File — Contains Fig A, Fig B. (DOCX) [file pone.0173245.s001.docx]
